# Loss of *vtx* Genes after the First Subcultivation Step of Verocytotoxigenic *Escherichia coli* O157 and Non-O157 during Isolation from Naturally Contaminated Fecal Samples

**DOI:** 10.3390/toxins3060672

**Published:** 2011-06-20

**Authors:** Maria-Adelheid Joris, Karen Verstraete, Koen De Reu, Lieven De Zutter

**Affiliations:** 1 Ghent University, Faculty of Veterinary Medicine, Salisburylaan 133, 9820 Merelbeke, Belgium; Email: lieven.dezutter@ugent.be; 2 Institute for Agricultural and Fisheries Research, Technology and Food Science Unit, Brusselsesteenweg 370, 9090 Melle, Belgium; Email: karen.verstraete@ilvo.vlaanderen.be (K.V.); koen.dereu@ilvo.vlaanderen.be (K.D.R.)

**Keywords:** *Escherichia coli*, VTEC, loss, *vtx* genes

## Abstract

Verocytotoxins VT1 and VT2,produced by Verocytotoxigenic *Escherichia coli* (VTEC), are encoded on temperate bacteriophages. Several studies reported the loss of the *vtx* genes after multiple subcultivation steps or long preservation. The objective of this study was to determine if the loss of the verocytotoxin genes can already occur during the first subcultivation step. Consequently, the stability of the *vtx* genes were tested in 40 isolates originating from 40 *vtx*-positive fecal samples after the first subcultivation step following the isolation procedure. The loss occurred in 12 out of 40 strains tested and was rather rare among the O157 strains compared to the non-O157 strains. This is the first study demonstrating that the loss of the verocytotoxin genes can already occur after the first subcultivation step. This may lead to an underestimation of VTEC positive samples.

## 1. Introduction

Verocytotoxigenic *Escherichia coli* (VTEC), also referred to as Shiga toxin-producing *E. coli* (STEC), are zoonotic pathogens associated with a high variety of clinical outcomes such as diarrhea, hemorrhagic colitis (HC) and hemolytic uremic syndrome (HUS). Human infections are in most cases acquired through water or food directly or indirectly contaminated with cattle feces. The key virulence factors are the verocytotoxins. There are two main types, namely VT1 and VT2, which can be further divided into subtypes based on their sequence analysis. The nomenclature is not definite and new variants are constantly being described. These VT-encoding genes (*vtx*) are generally encoded by a heterogenous group of temperate lambdoid bacteriophages and are expressed when the lytic cycle is activated [1,2]. 

In a cross-sectional survey, both *vtx*-negative and *vtx*-positive strains belonging to the same serogroup were detected on two cattle herds [3]. The absence of *vtx* genes in strains carrying *eae* and EHEC-hlyA is thought tooccur by two hypotheses. First of all, the *vtx*-negative strains could arise from strains that had lost their *vtx* genes during subculturing as several studies reported the spontaneous loss of *vtx* genes after multiple subcultivation steps [4,5,6,7,8]. Secondly, these *vtx*-negative strains could occur as inherently *vtx*-negative strain in the animal reservoir as such [9], designated as aEPEC.

Since several studies observed the loss after multiple subcultivation steps and long preservation times, this study was set up to determine (i) if the loss of *vtx* genes can already occur after the first subcultivation step after isolation, (ii) the frequency of this spontaneous loss, and (iii) to evaluate the toxin-type and serogroup dependence. 

## 2. Materials and Methods

Rectal fecal samples were taken from cattle known to be infected with VTEC O157 and/or non-O157, on 3 cattle farms, to study the spontaneous loss of *vtx*genes after the first subcultivation step of suspected colonies.

### 2.1. VTEC O157

To isolate *E. coli *O157 from fecal samples, 225mL of modified TSB, supplemented with 0.25 mL novobiocin was added to 25 g of feces. After incubation for 6h at 42 °C, immunomagnetic separation technique (IMS) using specific Dynabeads (Invitrogen, Paisley, UK) was performed according the manufacturer’s recommendations. The resulting suspension was plated onto cefixime-tellurite sorbitol-MacConkey agar (Oxoid Ltd., London, UK) and incubated overnight at 42 °C. From each sample one well isolated suspected colony was transferred to tryptone soy agar (Oxoid) and incubated for 24h at 37 °C. Subsequently, 10 colonies of each subculture on TSA were examined for the presence of virulence genes by a multiplex PCR, applying the primers for *vtx1*, *eae *and EHEC-*hlyA* described by Fagan *et al.*[10] and for *vtx2*described by Paton [11]. One isolate from the subculture was further tested for agglutination with an *E. coli* O157 latex test kit (Oxoid) for serogroup O157 confirmation.

### 2.2.VTEC Non-O157

For the isolation of EHEC non-O157, the isolation method described by Possé [12] was used. Briefly, a 25 g amount of each sample was enriched during 24 h at 42 °C in 225 mL tryptone soya broth (TSB) supplemented with 8 mg L^−1^ novobiocin, 16 mg L^−1^ vancomycin, 2mg L^−1^ rifampicin, 1.5 g L^−1^ bile salts and 1.0 mg L^−1^ potassium tellurite. After 6 and 24 h of incubation, respectively,100 and 10 µLof the enrichment broth was plated onto the new differential agar medium for O26, O111, O103 and O145[13]. Besides this direct plating, IMS was applied after 24 h on the enrichment broth. For the serogroups O26 and O103, Dynabeads (Invitrogen) were used, whereas for the serogroups O111 and O145, Captivate beads (Lab M, Lancs, UK) were applied. Afterwards, 100 µL of the IMS suspension was also plated onto these differential agar media. From each sample, one well isolated colony with a suspected morphology wassubcultured to trypton soy agar (TSA) (Oxoid). Subsequently, 10 colonies of each subculture on TSA were examined for the presence of virulence genes by a multiplex PCR, as described above. Subsequently serogroup-specific PCR was conducted for the serogroups O26, O103, O111 and O145 [14].

## 3. Results and Discussion

Enterohemorrhagic *E. coli * (EHEC) are a distinct class of VTEC, characterized by the presence of verocytotoxins, intimin and EHEC enterohemolysin. The key virulence determinants are the verocytotoxins 1 and 2 encoded on temperate bacteriophages. EHEC strains may convert to atypical enteropathogenic *E. coli* (aEPEC) strains by the loss of their *vtx* genes after multiple subcultivation steps or long preservation. These potential genetic changes of the pathogens have to be taken into account when interpreting screening results for the public health concern of O157 and non-O157 *E. coli*. To our knowledge, this is the first study conducted to examine the frequency of the loss of *vtx* genes after the first subcultivation step among O157 and non-O157 EHEC strains during the isolation from naturally contaminated bovine fecal samples, as other studies focused on multiple subcultivation steps and long-term preservation. In this survey, fecal samples were collected on three farms housing EHEC carrier animals. The stability of *vtx* genes were tested in 40positive fecal samples (20 O157 and 20 non-O157 samples) after the first subculturing step. Noteworthy, this subcultivation step is advisable and even obligatory by the ISO method 16654:2001 [15] for the detection of *E. coli* O157 from feed and food to ensure the purity of the strains to be characterized.

**Table 1:** Spontaneous loss of *vtx*genes after the first subcultivation step among EHEC O157 and non-O157 strains.

**Serogroup**	**N° of Strains**	***Vtx *Genes**			**Spontaneous Loss of *Vtx*Genes**		
		*Vtx1*	*Vtx2*		*Vtx1*	*Vtx2*	Range (1–10)
O157	20	20	20		1	2	1–2
Non-O157							
O26	3	3	1		2	0	2–3
O103	2	2	0		2	0	1–3
Non-Typed	15	15	3		3	2	1–8
Total	40	40	24		8	4	1–8

All tested strains were *eae* and EHEC-*HlyA* positive. The loss of *vtx* genes among O157 strains was rather rare compared to non-O157 strains, namely in 3 out of 20 O157 strains compared to 9 out of 20 non-O157 strains.Regardless of the differences in the isolation procedures, the frequency of this curing within the 3 O157 strains was rather rare (on average 1 out of 10), while for the 9 non-O157 strains, the rate of loss was higher (on average 4 out of 10 isolates). These findings are in agreement with Schmidt *et al.*[16] who observed that *vtx* genes appear to be more stably maintained in O157 strains than in non-O157. Because at least one colony still harbored *vtx1* and/or *vtx2* after subcultivation, it can be hypothesized that the loss already occurred during growth on the differential media. Our data may be of importance in the screening of VTEC because these toxigenic organisms of public health concern can become non-toxigenic after subcultivation. The question remains how frequently the loss of *vtx* genes already occurs in the intestine of cattle as Bielaszewska *et al*. [17] reported that an appreciable subset of patients suffering from HUS excreted EHEC that lost their *vtx* genes. The latter showed a common phylogeny with the EHEC of the corresponding serotype and they belong to the same MLST clonal complexes. The importance of free *vtx*-encoding bacteriophages should be addressed with regard to their role as vectors for horizontal virulence gene transfer [18] in the animal reservoir as Cobbaut [19] reported the presence of inherently *vtx*-negative strains on *vtx*-positive cattle herds. Therefore, the role of VT-producing *E. coli* may be of a greater concern than was previously assumed if there can be an alternate conversion between aEPEC and EHEC strains.

## 4. Conclusions

Our results provethat a loss of *vtx* genes in *vtx-*positive isolates can already occur after the first subculturing step of VTEC isolated from naturally contaminated samples. Consequently, this may lead to an underestimation of VTEC in animals, food and humans. Therefore, we advise to test different colonies instead of a single colony from a subculture for the presence of *vtx* genes in order to avoid false negative results.
